# Misdiagnosis of Small Cell Prostate Cancer: Lessons Learned

**DOI:** 10.7759/cureus.8356

**Published:** 2020-05-29

**Authors:** Dawood Findakly, Jue Wang

**Affiliations:** 1 Internal Medicine, Creighton University Arizona Health Education Alliance/Valleywise Health Medical Center, Phoenix, USA; 2 Genitourinary Oncology, Creighton University School of Medicine/University of Arizona Cancer Center at Dignity Health, Phoenix, USA

**Keywords:** small cell carcinoma of the prostate, diagnostic error, misdiagnosis, delayed diagnosis, surgical pathology

## Abstract

Small cell carcinoma (SCC) of the prostate is rare in the spectrum of prostate tumors. Clinically, prostate SCC is more aggressive and has a poorer prognosis when compared to prostate acinar adenocarcinoma. Delay in diagnosis and misdiagnosis further worsens the outcome of this disease. Here we present a 68-year-old man whose prostate SCC was initially misdiagnosed with benign prostate hypertrophy (BPH) by his primary care physician (PCP) and urologist and small cell lung cancer by his first oncologist and pathologist. This case underscores the diagnostic conundrum of prostate SCC and the importance of exercising caution against the possibility of uncommon prostate cancer misdiagnosis in clinical practice.

## Introduction

Diagnostic errors in oncology are potentially detrimental to patients, yet such errors remain understudied. Accurate diagnosis is the first step for the successful management of cancer. Globally, prostate cancer is the third most common cancer and the sixth leading cause of cancer mortality among men [1]. Pure prostate small cell carcinoma (SCC) constitutes a rare and aggressive subtype of prostate cancer with a higher mortality rate given it usually presents at a more advanced stage with visceral and lytic bone metastasis [2,3]. The initial presenting symptoms of prostate SCC are similar to those of adenocarcinoma; hence, it is often misdiagnosed. We present a particular case of advanced stage SCC of the prostate in a 68-year-old man who was misdiagnosed initially as benign prostate hypertrophy (BPH) and SCC of the lung and discuss the hurdles in the diagnosis and management of patients with prostate SCC.

## Case presentation

A 68-year-old non-smoking Caucasian man initially transferred from a different hospital for evaluation of a brain lesion found on imaging. The patient has a medical history pertinent to BPH, and his family history was relevant for a father with colon cancer.

Six months earlier, his primary care physician (PCP) evaluated him for worsening urinary symptoms and subsequently referred him to a urologist. Upon further evaluation, his prostate-specific antigen (PSA) level was normal at 3 ng/mL, and his digital rectal examination was normal, for which a cystoscopy with prostate GreenLight™ (Boston Scientific, Marlborough, MA) enucleation was performed for BPH diagnosis. Five months later and one month before this admission, he discerned progressive worsening of double vision. His PCP examined and referred him to an ophthalmologist, who diagnosed him with third cranial (oculomotor) and sixth cranial (abducens) nerve palsy, which was subsequently confirmed by a neurologist.

Subsequently, the patient was admitted to the other hospital, and upon admission, he was found to have an acute kidney injury (AKI) and hyperkalemia. Laboratory studies showed white blood cells of 27 x 10^3/µL, hemoglobin of 11.5 g/dL, and platelet count of 201 K/µL. His chemistry was pertinent for sodium of 134 mmol/L, potassium of 6.5 mmol/L, chloride of 103 mmol/L, bicarbonate level of 10 mmol/L, a glucose of 159 mg/dL, creatinine of 13 mg/dL, uric acid of 19.6 µmol/L, chromogranin A of 656 µg/L, and PSA of 22.67 ng/mL. He received hemodialysis and underwent bilateral percutaneous nephrostomy, after which his AKI resolved. An abdominal CT scan showed findings consistent with diffuse metastatic disease to the lungs, liver, and peritoneum, and brain MRI showed brain lesions. Therefore, he was, subsequently, transferred to our hospital for further care.

**Figure 1:**
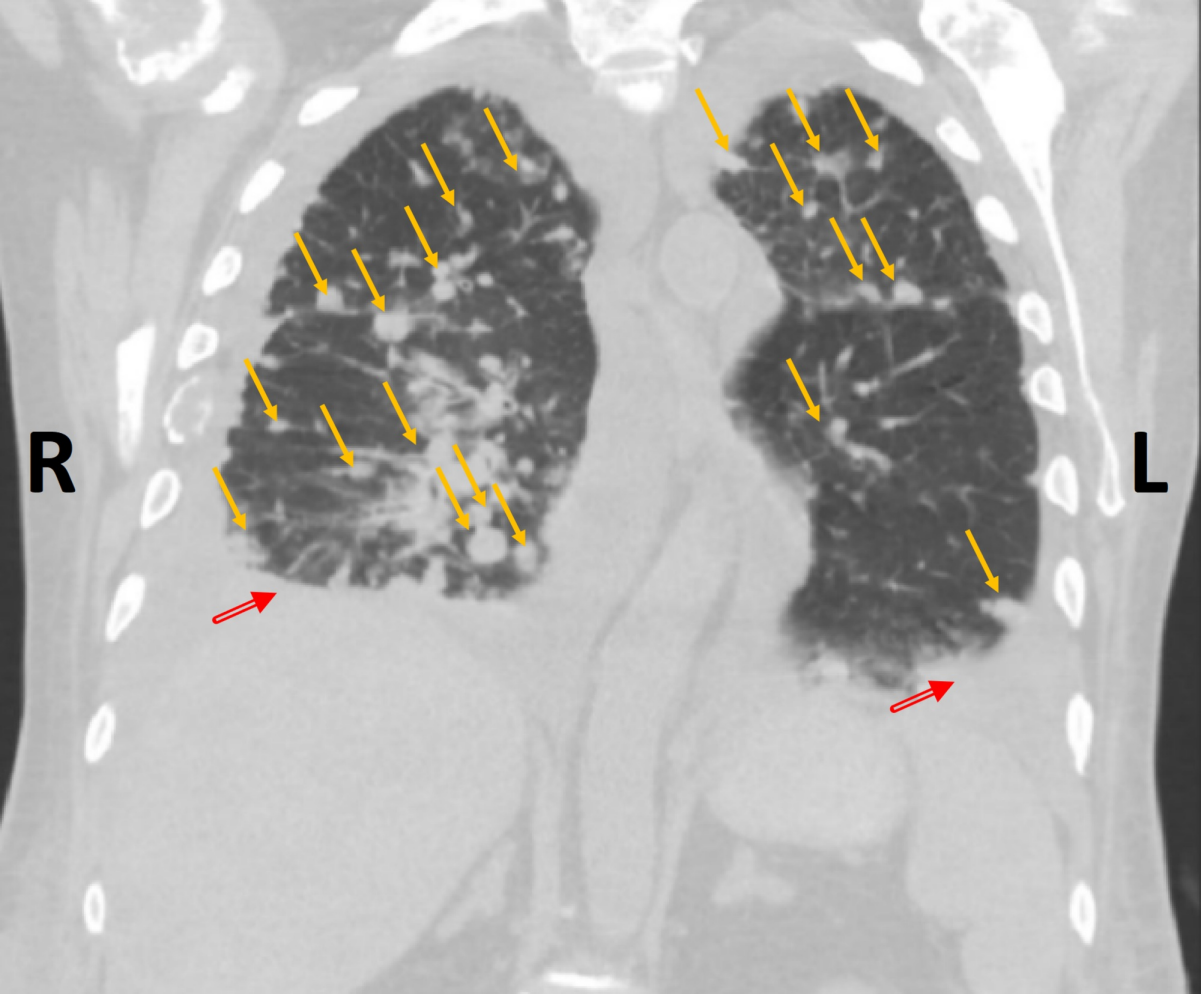
Coronal chest CT image, using lung window, showing multiple bilateral randomly distributed pulmonary nodules (yellow arrows) with bilateral pleural effusions (red arrows; R>L) R: right; L: left

**Figure 2:**
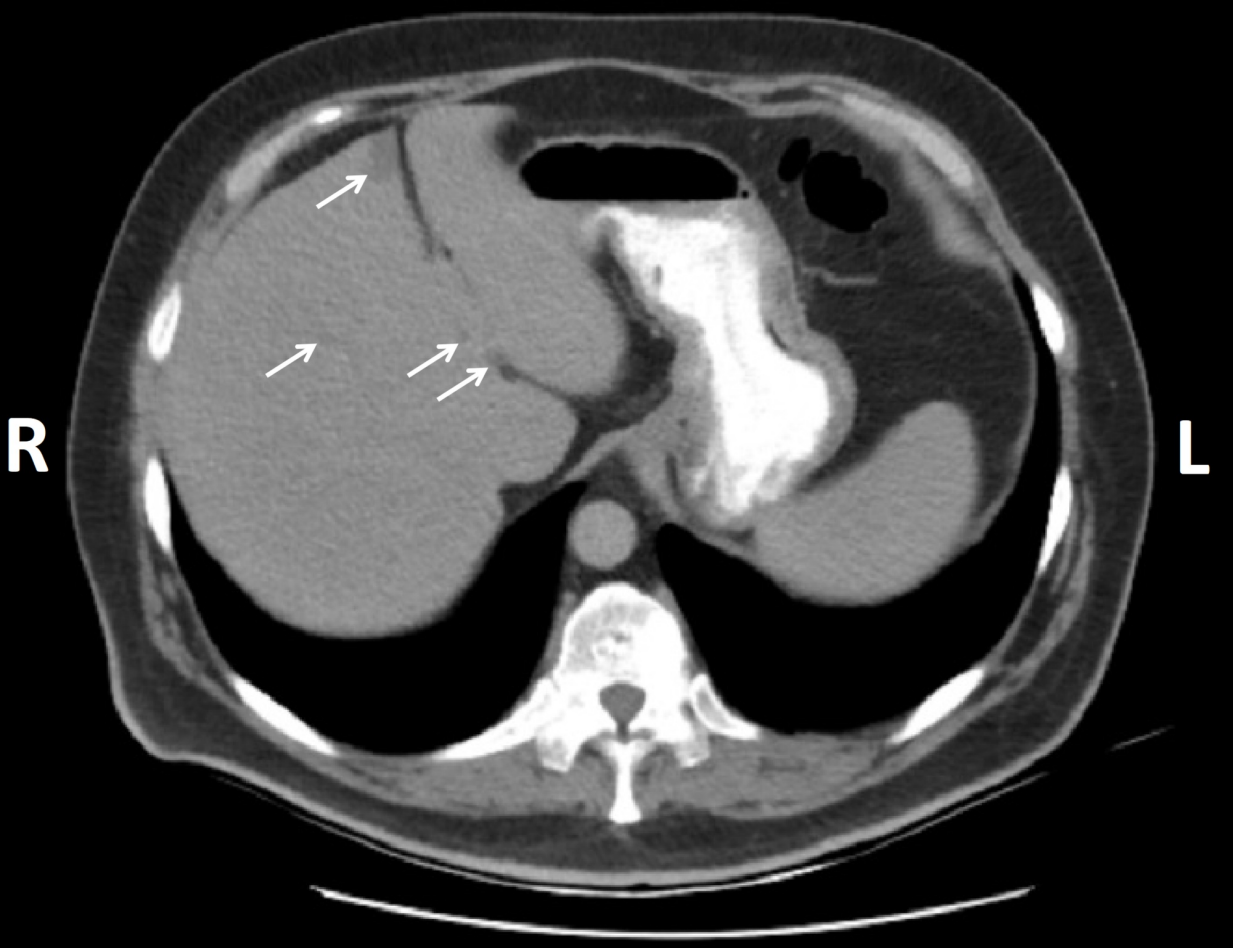
Abdominal CT scan showing hepatic focal hypodense hepatic lesions (white arrows) R: right; L: left

**Figure 3:**
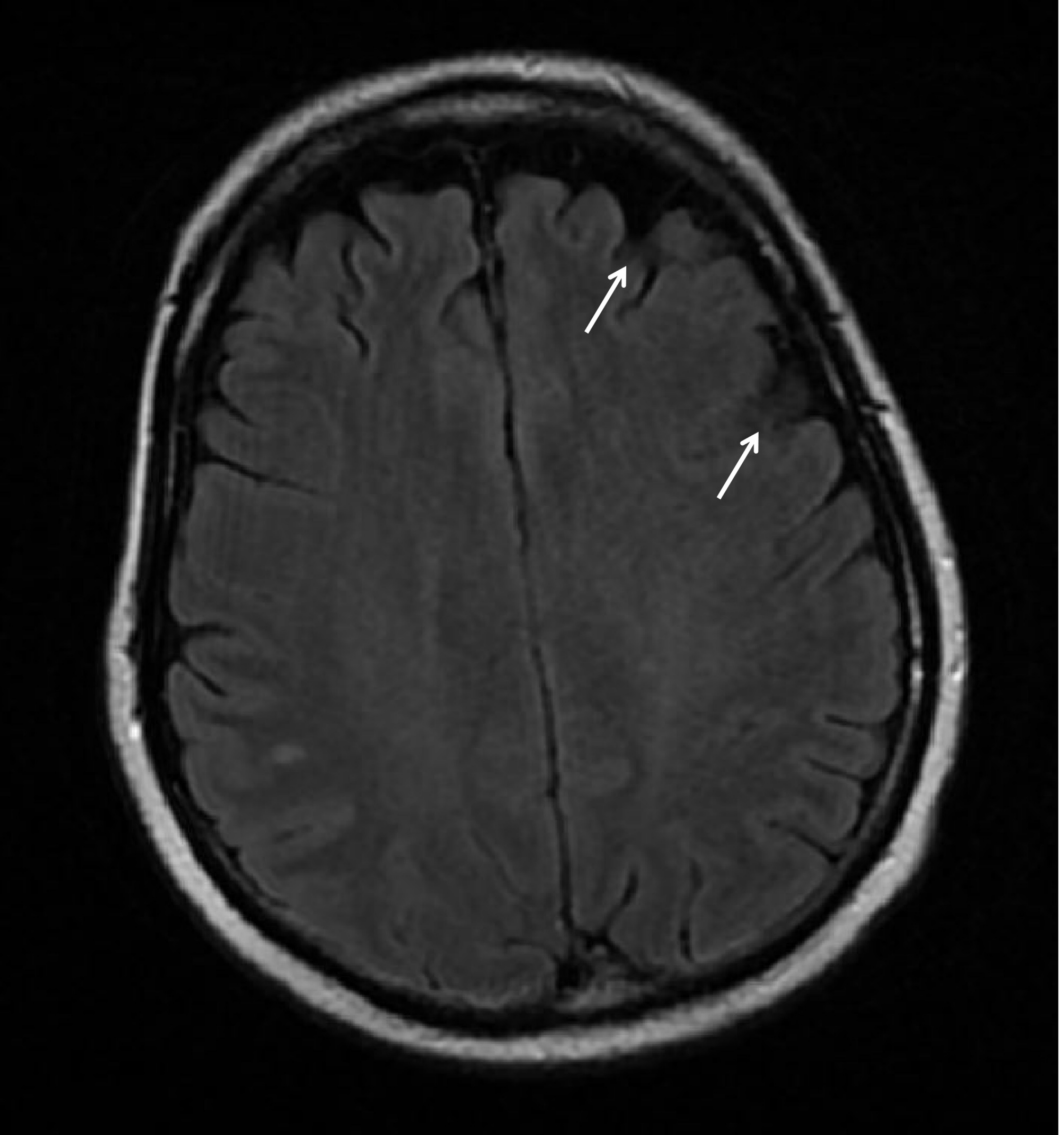
Axial MRI of the brain showing hypointense left frontoparietal lesions (white arrows) without midline shift

Upon evaluation in our hospital, imaging showed findings consistent with earlier recognized lesions at the other hospital (Figures 1-3). The patient was, therefore, seen by a multidisciplinary team of a neurosurgeon, an ophthalmologist, a medical oncologist, and a radiation oncologist. Biopsies of a perirectal mass showed a high-grade neuroendocrine tumor. The oncologist who saw him diagnosed him with metastatic SCC of the lung; therefore, he was started on one cycle of carboplatin/etoposide chemotherapy during his hospitalization, which he tolerated well and was discharged home. 

**Figure 4:**
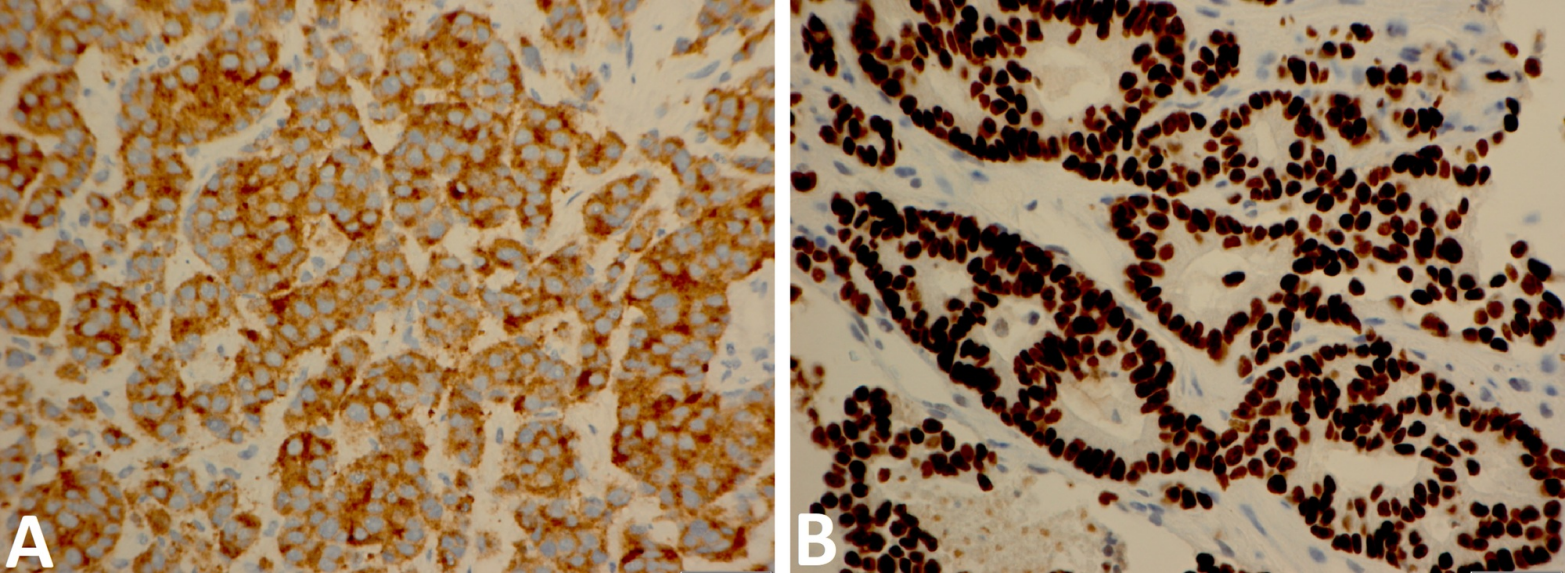
IHC for (A) synaptophysin and (B) TTF1 were performed on the samples derived from urethral tissue (x440 magnification) IHC: immunohistochemistry; TTF-1: thyroid transcription factor 1

A senior oncologist saw the patient, reviewed all his radiographic images, and questioned the initial diagnosis of lung SCC based on the patient’s clinical presentation of urinary obstruction, hydronephrosis, and AKI. Hence, the original tumor location speculated to be consistent with a high-grade neuroendocrine tumor/SCC originating from the urinary tract. The senior oncologist, therefore, advised cystoscopy for prostate biopsy, which was declined by consulting urologist because the “prostate exam was normal.” Consequently, the senior oncologist sent the patient to a second urologist who performed cystoscopy and informed the patient that everything was normal. The senior oncologist had a thorough discussion to explain the necessity of a meticulous endoscopic urethral examination and prostate biopsy. Subsequently, the patient took the senior oncologist’s advice, went back to the urologist, and underwent a repeat diagnostic cystoscopy. This time, biopsies of the urethra were taken and confirmed a high-grade large cell neuroendocrine carcinoma. The tissue specimen from this case was compared to the patient’s previous perirectal mass biopsy and was found to have similar features. Immunohistochemical (IHC) stains were negative for CK7, CK20, PSA, and prostate-specific acid phosphatase. Scattered individual cells were positive for nuclear NKX 3.1 and cytoplasmic CK5. Moreover, there was diffuse variable positivity for nuclear p63 with greater than 80% of nuclei were positive for p53 and diffuse strong positivity for CD56, thyroid transcription factor 1 (TTF-1), and synaptophysin (Figure 4).

**Figure 5:**
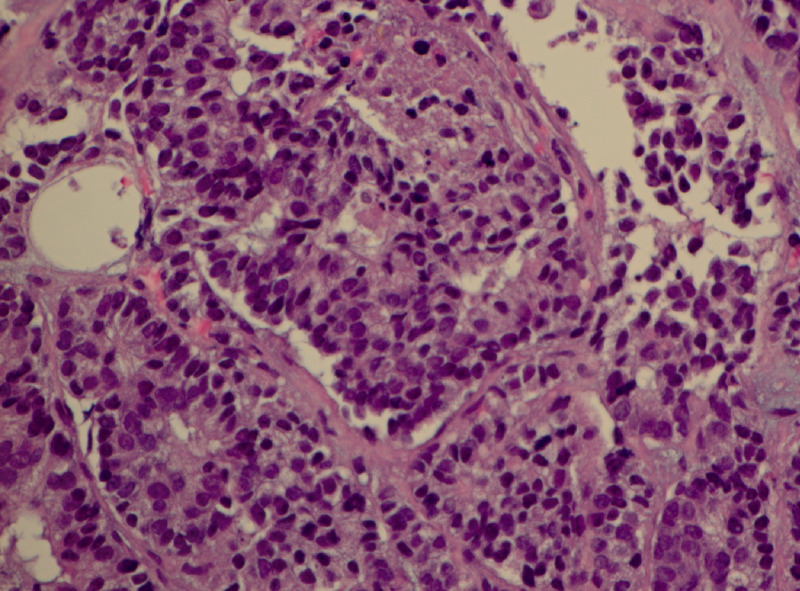
H&E staining performed on the samples derived from prostate tissue revealing prostate SCC (x400 magnification) H&E: hematoxylin and eosin; SCC: small cell carcinoma

Based on the clinical history of disease distribution, the pathologist considered the neoplasm to be likely of either prostate or urinary bladder origin. Subsequently, the senior oncologist reviewed the urethral biopsy, remained suspecting the primary site of cancer to be originating from the prostate. Therefore, he advised for prostate biopsy. Finally, the patient underwent transrectal ultrasound-guided prostate biopsy, which confirmed that all 12 cores were positive for prostate SCC (Figure 5). The pathology findings confirmed the senior oncologist’s initial surmise that the metastatic cancer origin was from the genitourinary tract, likely involving prostate extending into the urethra. 

## Discussion

Prostate SCC, first described by Wenk et al. in 1977, is a rare distinct clinical and pathologic entity, constituting approximately <1% of prostate cancer cases [4,5]. Patients with prostate SCC most frequently present with voiding, neurological (such as confusion and sensory or motor deficits), or constitutional symptoms and are generally associated with an abysmal prognosis [6]. Pathologically, SCC is primarily characterized by tumor cells that are usually monomorphic and arranged in uniform round hyperchromatic nuclei with salt-and-pepper pattern chromatin [7]. IHC stains are needed for small cell cancer diagnosis, and the panel should include chromogranin A, Ki67, and synaptophysin [8]. The clinical features of prostate SCC are characterized by rapid disease progression, low PSA relative to disease burden, lytic bone lesions with visceral metastases, and unresponsiveness to hormonal therapy [9]. Prostate SCC is highly aggressive as compared to stage-matched prostate adenocarcinoma, and it is essential to differentiate the two [10]. Unfortunately, misdiagnosis and delayed diagnosis contribute to the poor outcome of cancers, especially rare cancers, as it increases the risk for disease progression, which potentially affect outcomes because the medical treatment necessary to eradicate a later stage of the malignancy can be significantly more difficult for a patient to endure [11,12]. Diagnostic errors involve several types of missed opportunities to make a correct and timely diagnosis. Delayed and misdiagnosis can lead to harm from delayed or inappropriate treatments. The diagnosis and management of rare tumors are challenging as practitioners infrequently see them, and most data emanate from individual case reports and small single institutional series, and, therefore, fewer experts are focused on studying them. Prostate SCC is one of the most aggressive subtypes of prostate cancer. Early and accurate diagnosis is critical for a better outcome. A comprehensive history, physical examination, ordering appropriate workup, and consulting when in a dilemma are essential in identifying means to solve this intricacy and perpetrate actions through consistency in implementing those means to achieve improved outcomes. Prostate SCC is morphologically comparable to SCC of the lung which has led to misdiagnosis by pathologists and oncologists. In this case, the underlying potential reasons for initial misdiagnosis include an inadequate history, physical examination, failure to order and correctly interpret radiographic pathologic findings, and failure to get a consultation with a genitourinary oncologist. The reasons mentioned earlier contributed to the misdiagnosis of our patient at the PCP and urology office. The misleading clinical diagnosis resulted from the unfamiliarity with non-pulmonary SCC, such as prostate SCC, given it is a distinct entity to both urologists and general oncologists. Retrospectively, an initial transurethral resection of the prostate and pathological examination would have been possible in this case to provide an accurate assessment and to avoid the misdiagnosis. Even though the patient was a non-smoker and CT scan showed no dominant lesion, the oncologist did not doubt his presumptive diagnosis. Therefore, we underline the important, challenging clinical aspects in establishing the diagnosis of prostate SCC. In our case, the PCP and oncologist reached their erroneous diagnosis based on their incomplete assessment. In order to avoid misdiagnosis of rare cancers, such as prostate SCC, effective communication among multidisciplinary cancer care team members, PCP, urologist, and pathologist is essential. Despite the advanced technology, clinical history and physical examination remain the critical components of correct diagnosis and often provide more information than broad testing approaches. Performing a complete history and physical examination, and re-examining pathology slides, when in doubt, ask for expert opinion, all of these would help guide physicians to the correct diagnosis no matter how unachievable it might sound, to change the way physicians practice. Diagnosis is the foundation of medicine. Without the right diagnosis, patients do not receive the right treatment. Some diagnostic uncertainty is inevitable in the setting of a rare disease. The key to the correct diagnosis does not rely only upon expertise because some diagnostic uncertainty is inevitable. A productive communication between multidisciplinary team members and thoughtful coordination of follow-up care in a tertiary cancer center with particular expertise with this disease is critical in providing high-quality care.

## Conclusions

Clinicians should maintain a high index of suspicion for the possibility of cancer in patients presenting with prostate symptoms. Timely and complete evaluation of the unique clinical presentation of each patient is warranted, and when in doubt, it is better to revise the histological and immunohistochemical examination. In our institution, we are developing a protocol to enhance effective communication between multidisciplinary team members to reduce diagnostic error and promote a culture of diagnostic safety.
